# The Clinical Trials Landscape of Transcutaneous Electrical Acupoint Stimulation (TEAS) in Perioperative Care: Current Status and Future Perspectives

**DOI:** 10.7759/cureus.111093

**Published:** 2026-06-18

**Authors:** Yilong Liu, Jingjing Chen, Ying Liu, Beifang Ning, Kai Ding

**Affiliations:** 1 Gastroenterology Department, Changzheng Hospital, Naval Medical University, Shanghai, CHN; 2 Health Management Center, The Second Affiliated Hospital of Naval Medical University, Shanghai, CHN; 3 Emergency Intensive Care Unit, The Second Affiliated Hospital of Naval Medical University, Shanghai, CHN; 4 Gastroenterology Department, The Second Affiliated Hospital of Naval Medical University, Shanghai, CHN

**Keywords:** clinical trials landscape, gastrointestinal dysfunction, perioperative care, surgery, transcutaneous electrical acupoint stimulation

## Abstract

Transcutaneous electrical acupoint stimulation (TEAS) is a promising, non-invasive, non-pharmacological adjunct for perioperative care, yet a global overview of its clinical trial landscape remains lacking. This study systematically analyzed 239 perioperative TEAS trials (2012-2025) from three major registries (ClinicalTrials.gov, Chinese Clinical Trial Register (ChiCTR), and EU Clinical Trials Register (EU-CTR)). The number of registered trials has grown notably in recent years, with a clear upward trend observed in 2024. Research attention remains heavily concentrated on adult populations, while pediatric applications are comparatively understudied. Digestive system surgeries represent the most common clinical context, and primary outcome measures predominantly focus on symptomatic improvements related to gastrointestinal function and pain relief, with limited emphasis on mechanistic exploration. Furthermore, the majority of trials are either ongoing or yet to commence, orthopedic applications remain underexplored, and substantial heterogeneity exists across current trial designs. We identify critical research gaps and emphasize the urgent need for standardized large-scale randomized controlled trials (RCTs), pediatric studies, mechanistic exploration, and diversification across surgical specialties. These efforts may help establish TEAS as a potential complementary perioperative therapy alongside pharmacological interventions.

## Editorial

Over the past decade, perioperative medicine has shifted from an organ-centric to a patient-centric paradigm, with increasing emphasis on non-pharmacological adjunct therapies for their potential to enhance recovery and reduce opioid exposure [1]. Among these, transcutaneous electrical acupoint stimulation (TEAS), a non-invasive alternative to electroacupuncture, has demonstrated promise in mitigating postoperative nausea and vomiting (PONV), promoting gastrointestinal motility, and alleviating pain [2-4]. Acupuncture is an ancient form of Oriental medicine. TEAS combines the technical characteristics of acupuncture and transcutaneous electrical nerve stimulation, and uses surface electrodes to act on the adjacent or overlapping acupoints of nerves to exert a therapeutic effect. Furthermore, TEAS offers advantages such as flexible acupoint selection, non-invasiveness, safety, simplicity, and portability, and significantly improves patient compliance [2,3].

Despite the surge in exploratory trials, a comprehensive overview of the global perioperative TEAS research landscape is lacking. To address this, we systematically searched three major clinical trial registries (ClinicalTrials.gov, Chinese Clinical Trial Register (ChiCTR), and EU Clinical Trials Register (EU-CTR)) from their inception up to June 13, 2025, to characterize the trial populations, surgical contexts, outcome priorities, and knowledge gaps. The search terms were "Transcutaneous acupoint electrical stimulation", "Transcutaneous Electrical Acustimulation", and "Transcutaneous electrical acupoint stimulation". The inclusion criteria were as follows: 1) the intervention was clearly defined as TEAS; 2) participants were surgical patients with no restrictions on age or gender; 3) TEAS was administered during the perioperative period (TEAS applied preoperatively, intraoperatively, or postoperatively in patients undergoing any surgical procedure). The exclusion criteria were as follows: 1) Trials without TEAS intervention, or those applying TEAS in non-perioperative settings; 2) Duplicate registrations of the same study across multiple databases (only the latest and most complete record was retained); 3) Registrations with severely missing information that prevented identification of core study elements; 4) Non-interventional studies, including observational cohorts, case reports, and secondary analyses. All registered records were independently screened manually by two researchers: duplicate entries were first removed (duplicates across the three registries were manually identified and removed by comparing trial titles, interventions, study conditions, and investigator information; only unique trials were retained), followed by one-by-one verification of registration details. Discrepancies were resolved through discussion, and arbitration by a third researcher was sought when necessary. Data statistics and figure generation in this study were performed using Microsoft Excel 2021 (Microsoft Corporation, Redmond, WA, US), GraphPad Prism 9 (GraphPad Software, Boston, MA, US), and R software (version 4.3.1; R Core Team (2023)).

**Figure 1:**
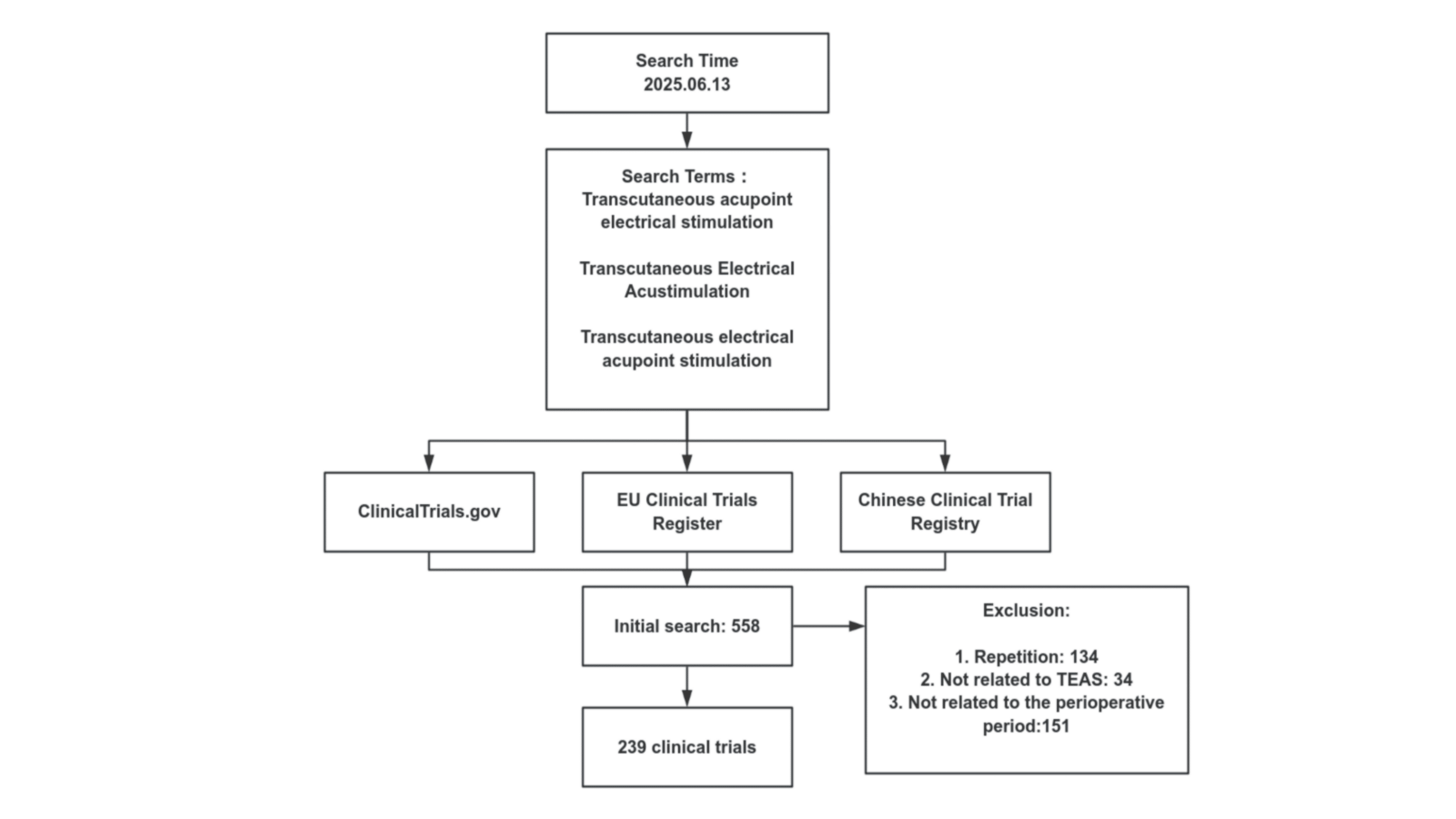
Flowchart of the study process

**Figure 2:**
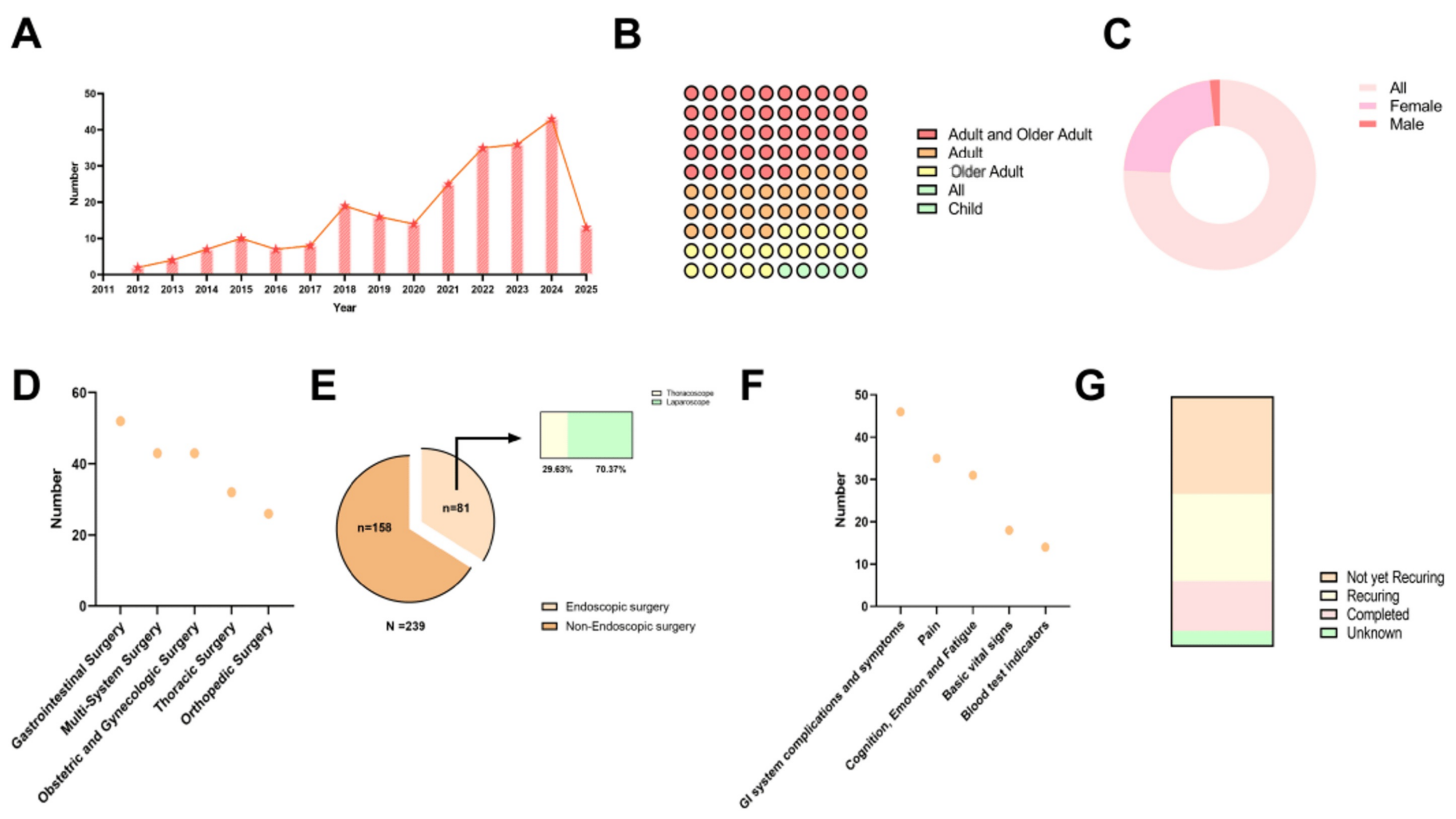
Overview of clinical trials of TEAS in perioperative care (A) Annual distribution of clinical trials (2012-2025). (B) Distribution of clinical trials by the age of the trial population (adult:18-64 years, older adult: ≥65 years, child:＜ 18 years). (C) Distribution by gender. (D) Top 5 surgical categories. (E) Distribution of trials by endoscopic surgery. (F) Top five primary outcomes of digestive surgery. (G) Status distribution of trials.

A total of 239 perioperative TEAS trials were retrieved between 2012 and 2025 (Figure 1). Prior to 2020, annual trial numbers grew modestly, followed by a sharp increase in 2024 (n=43, Figure 2A), including trials across different statuses, including ongoing, completed, and terminated studies. This acceleration may be attributable to the growing research interest in the application of TEAS within perioperative care. Concurrently, this inflection point coincided with the later stages of enhanced recovery after surgery (ERAS) protocol dissemination and the urgency for opioid-sparing strategies, suggesting TEAS may be transitioning from fringe exploration toward mainstream adjunct therapy [5].

Trial populations were highly concentrated in adults, comprising 94.98% (227/239) of the studies (Figure 2B). Only 5.02% included pediatric patients (<18 years), reflecting possible ethical and technical barriers that may contribute to the limited number of pediatric trials. These potential challenges include lower surgical volumes in children, stringent informed consent requirements, and age-dependent variations in acupoint size. Moreover, only 24.27% (58/239) of the trials imposed specific gender restrictions on participants, indicating an underexplored opportunity for research on TEAS dose individualization based on sex hormones (Figure 2C).

Digestive system surgeries dominated the included perioperative studies (21.76% of the total trials) (Figure 2D). Gastric (n=15), intestinal (n=12), and combined gastrointestinal surgery (n=12) were the top three subcategories (Table 1). Surgeries involving the hepatobiliary-pancreatic system and esophagus accounted for only 25.0% (13/52) of digestive system trials, despite these procedures also facing significant perioperative complications, such as gastrointestinal dysfunction and pain (Table 2). Thus, perioperative management of these organs warrants greater research focus. Notably, 42.31% (22/52) of digestive system trials involved tumor resection, implying that patients with cancer may present more complex perioperative challenges, potentially related to preoperative medications, poorer general health, and nutritional status (Table 3) [6]. Consequently, researchers anticipate greater benefits of TEAS in facilitating gastrointestinal recovery in these patients. Approximately 66.11% (158/239) of the included surgeries were non-laparoscopic and potentially associated with longer operative times, greater tissue trauma, and higher anesthetic doses, leading to increased perioperative adverse events and a greater need for effective recovery aids (Figure 2E).

**Table 1:** Subgroups of digestive surgery

Operative site	Number
Stomach	15
Intestinal tract	12
Stomach and intestinal tract	12
Gall bladder	5
Liver	4
Bile duct	1
Pancreas	1
Stomach and esophagus	1
All	1
Tumor-associated	
Yes	22
No	30

**Table 2:** Clinical research outcomes related to digestive system surgery It should be noted that postoperative nausea, postoperative vomiting, and combined postoperative nausea and vomiting were recorded separately. This classification strictly follows the original outcome settings of each registered trial, as different studies adopted distinct indicator forms.

Outcome	Number
Postoperative nausea and vomiting	29
The time of the first postoperative defecation	7
Postoperative nausea	5
Postoperative intestinal obstruction	3
The first intestinal peristalsis after the operation	3
The time of the first postoperative bowel movement	3
Postoperative vomiting	1
Postoperative gastrointestinal function	1
Overall symptoms of the digestive tract	1

**Table 3:** Known status of clinical trials on TEAS in perioperative care All percentages were calculated based on the total number of trials within each corresponding subgroup. Trials labeled as withdrawn, suspended, or terminated were not excluded from the present analysis. TEAS: transcutaneous electrical acupoint stimulation

	Completed, n (%)	Recruiting, n (%)	Not yet Recruiting, n (%)
Type of Surgery			
Gastrointestinal Surgery	8 (16.33)	20 (40.82)	21 (42.86)
Multi-system Surgery	13 (32.50)	16 (40.00)	11 (27.50)
Obstetric and Gynecologic Surgery	10 (23.26)	16 (37.21)	17 (39.53)
Thoracic Surgery	5 (17.24)	13 (44.83)	11 (37.93)
Orthopedic Surgery	2 (8.70)	7 (30.43)	14 (60.87)
Primary Outcome			
GI System Complications and Symptoms	6 (12.00)	19 (38.00)	25 (50.00)
Pain	4 (9.76)	15 (36.59)	22 (53.66)
Cognition, Emotion, and Fatigue	5 (13.89)	17 (47.22)	14 (38.89)
Basic Vital Signs	7 (30.43)	6 (26.09)	10 (43.48)
Blood Test Indicators	3 (20.00)	7 (46.67)	5 (33.33)
Related to Endoscopy			
Endoscopic Surgery	12 (15.79)	34 (44.74)	30 (39.47)
Non-endoscopic Surgery	36 (24.16)	50 (33.56)	63 (42.28)
Age			
Adult	14 (20.29)	21 (30.43)	34 (49.28)
Older Adult	7 (15.56)	20 (44.44)	18 (40.00)
Sex			
Female	18 (33.33)	15 (27.78)	21 (38.89)
All	30 (17.96)	68 (40.72)	69 (41.32)

The two most frequently assessed primary outcomes were gastrointestinal symptoms and pain (Figure 2F). Unsurprisingly, both surgical types and primary outcomes were related to the digestive system. Therefore, we analyzed the perioperative observation endpoints of the digestive system surgical trials. It turns out that postoperative nausea and vomiting were the most common endpoints (n=29), followed by assessments of intestinal motility recovery (e.g., time to first flatus/defecation, first bowel movement). Moreover, a few studies considered adverse events, such as postoperative ileus (n=3), as outcomes (Table 2). This focus contrasts sharply with trials in fields like non-alcoholic fatty liver disease (NAFLD), where metabolic surrogate markers (e.g., magnetic resonance imaging-proton density fat fraction (MRI-PDFF)) or histological non-alcoholic steatohepatitis (NASH) resolution are primary endpoints, highlighting the current emphasis on symptomatic relief over mechanism-driven efficacy validation in perioperative TEAS research [7].

**Figure 3:**
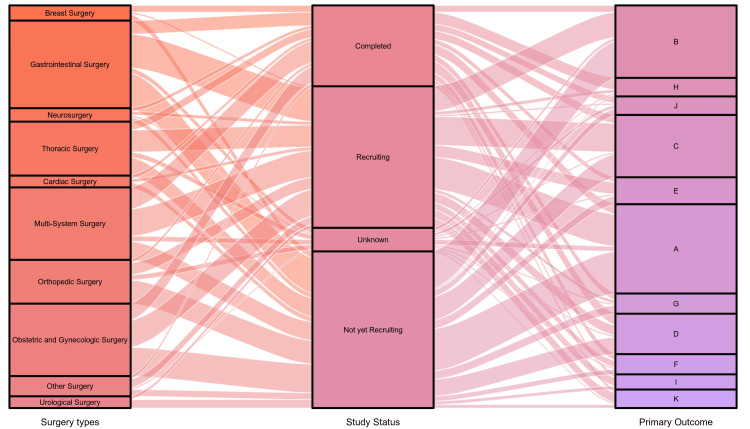
Sankey plots integrating the type of surgery, trial status, and primary outcome A: Digestive system-related symptoms, signs, and complications; B: Pain; C: Cognition, mood, and fatigue; D: Basic vital signs; E: Blood laboratory parameters; F: Health status or quality of life; G: Sleep; H: Anesthetic and analgesic medication usage; I: Respiratory system-related symptoms, signs, and test parameters; J: Complications and signs outside the digestive and respiratory systems; K: Others

Among trials with a known status, 35% were actively recruiting, 20% were completed, and 39% did not start recruitment (Figure 2G). The completion rates varied by specialty: gastrointestinal surgery (16.33%), obstetrics and gynecology (23%), and orthopedics (8.70%). Perioperative TEAS research in orthopedic surgeries (n=23) remains relatively scarce, even though postoperative gastrointestinal dysfunction is a common challenge, particularly following spinal surgery. This indicates a potential role for neuromodulation approaches in this clinical setting​​​​​​ [8]. A Sankey plot integrating surgery type, trial status, and primary outcome clearly illustrates that abdominal surgeries (gastrointestinal and gynecological), whether ongoing or planned, predominantly target gastrointestinal dysfunction (e.g., PONV) and pain as the primary endpoints (Figure 3, Table 3). This underscores the need for greater diversification in both the types of surgeries studied and the endpoints measured.

This study employed a novel approach for enhanced comprehensiveness: the inclusion of three prominent clinical trial registries, which is unprecedented among related studies. Despite the rapid growth in perioperative TEAS research, significant biases persist in the landscape: a predominance of gastrointestinal surgeries, a concentration on adult populations, and a methodological inclination towards symptoms rather than mechanisms. These are the key areas for improvement in future studies. Furthermore, substantial heterogeneity exists in current TEAS trial designs regarding acupoint selection, stimulation parameters, control interventions, and treatment regimens. There is an urgent need for high-quality, large-scale standardized randomized controlled trials (RCTs) to generate robust evidence. It is also noteworthy that many registered trials lack complete documentation of methodological details, including blinding, randomization, and sample size information. This will also help standardize TEAS clinical research protocols. Such efforts hold the promise of establishing TEAS as a "complementary non-pharmacological therapy" providing patients with a safe, non-invasive, and convenient therapeutic option to improve perioperative care quality and outcomes for more patients.
